# Association between Parkinson’s Disease and Psychosocial Factors: Results of the Nationally Representative German Ageing Survey

**DOI:** 10.3390/jcm11154569

**Published:** 2022-08-05

**Authors:** Regina Vardanyan, Hans-Helmut König, André Hajek

**Affiliations:** Department of Health Economics and Health Services Research, University Medical Center Hamburg-Eppendorf, Hamburg Center for Health Economics, 20246 Hamburg, Germany

**Keywords:** Parkinson’s disease, life satisfaction, optimism, loneliness, perceived social isolation, perceived autonomy

## Abstract

Objective: The aim of this study was to clarify the link between Parkinson’s disease (i.e., comparing individuals with Parkinson’s disease and individuals without Parkinson’s disease) and psychosocial outcomes (in terms of life satisfaction, optimism, loneliness, perceived social isolation and perceived autonomy). Methods: Cross-sectional data (wave 5) were used from the nationally representative German Ageing Survey (with *n* = 7832). Life satisfaction was quantified using the Satisfaction with Life Scale. Optimism was measured using the Brandstädter and Wentura tool. Perceived autonomy was quantified using the Schwarzer tool. Loneliness was quantified using the De Jong Gierveld tool. Perceived social isolation was quantified using the Bude and Lantermann tool. Physician-diagnosed Parkinson’s disease served as the key independent variable. Results: Multiple linear regressions showed that individuals with Parkinson’s disease reported significantly lower perceived autonomy (β = −0.30, *p* < 0.01) compared to individuals without Parkinson’s disease. In contrast, they did not report worse psychosocial outcomes (in terms of life satisfaction, optimism, loneliness and perceived social isolation). Conclusion: Study findings showed a quite strong association between Parkinson’s disease and perceived autonomy. Future research could elucidate the underlying mechanisms.

## 1. Introduction

Parkinson’s disease is a multifaced neurodegenerative disorder combining motor and nonmotor features. It can be defined as “a clinical syndrome dominated by a disorder of movement consisting of tremors at rest, rigidity, elements of slowness of movements (bradykinesia), reduced movements (hypokinesia), loss of movements (akinesia), and postural abnormalities.” [1].

Clinical management of Parkinson’s disease demands attention beyond its motor symptoms and requires a respective awareness of its nonmotor features (neuropsychiatric disturbances), such as depression, sleep abnormalities, anxiety and psychosis, as well as behavioral and cognitive changes. More precisely, depression is a key nonmotor symptom in Parkinson’s disease. Depression appears in the early stage and persists throughout the disease duration [2]. Moreover, a very recent systematic review and meta-analysis of 129 studies showed that the prevalence of depression in Parkinson’s disease was 38% [2].

As Parkinson’s disease is associated with depression, it must be treated in a timely manner, as otherwise, it may extend beyond mood symptoms and lead to faster physical and cognitive deterioration and poorer quality of life [3]. Parkinson’s disease is also linked with increased mortality—as shown by Macleod et al. in 2014 [4].

While various consequences of Parkinson’s disease are well-known (such as decreased longevity or decreased mental health), the psychosocial consequences of Parkinson’s disease are poorly understood. Actually, there is very limited knowledge regarding the association between Parkinson’s disease and psychosocial factors. For example, in a theoretical paper, Prenger et al. assumed that individuals with Parkinson’s disease may report high levels of loneliness and social isolation [5]. An empirical study by Jonasson et al. examined the determinants of life satisfaction among individuals with Parkinson’s disease (but without including a healthy control group) [6]. A further empirical study showed an association between low optimism and reduced quality of life among individuals with Parkinson’s disease (again, without including a healthy control group) [7]. Once more, only among individuals with Parkinson’s disease, another study reported low functional autonomy scores [8].

In sum, the large majority of previous studies failed to include a comparison group (i.e., individuals without Parkinson’s disease). Additionally, previous studies did not use data from nationally representative samples, but commonly used very small clinical samples. It is important to investigate the association between Parkinson’s disease (i.e., comparing individuals with Parkinson’s disease and individuals without Parkinson’s disease) and psychosocial factors in terms of life satisfaction, optimism, perceived autonomy, loneliness and perceived social isolation. Such understanding is important because these psychosocial factors can contribute to successful ageing. Life satisfaction refers to the “individual cognitive evaluation of life as a whole” [9]. Optimism can be defined as an “individual difference variable that reflects the extent to which people hold generalized favorable expectancies for their future” [10]. Perceived autonomy refers to the “capacity to think, decide, and act on the basis of such thought and decision freely and independently” [11]. Loneliness can be defined as a “distressing feeling that accompanies the perception that one’s social needs are not being met by the quantity or especially the quality of one’s social relationships” [12]. Perceived social isolation can be defined as the feeling that one does not belong to the society [13].

Thus, our aim is to clarify the association between Parkinson’s disease (i.e., comparing individuals with Parkinson’s disease and individuals without Parkinson’s disease) and psychosocial factors (in terms of life satisfaction, optimism, perceived autonomy, loneliness and perceived social isolation). This knowledge can potentially stress the importance of the reduced psychosocial well-being that is likely to be found among individuals with Parkinson’s disease in comparison to individuals without Parkinson’s disease due to factors possibly associated with Parkinson’s disease (such as perceived stigma, homebound-ness, lower self-esteem).

## 2. Materials and Methods

### 2.1. Sample

The sample of individuals was retrieved from the fifth (2014) wave of the German Ageing Survey (DEAS, “Deutscher Alterssurvey”). This nationwide, representative cohort-sequential study combines cross-sectional samples with longitudinal samples while relying on participants of the community-dwelling population aged 40 years and older in Germany. It is organized by the German Center for Gerontology (DZA, “Deutsches Zentrum für Altersfragen”), which was funded by the German Federal Ministry for Family Affairs, Senior Citizens, Women and Youth. The first wave took place in 1996, and following subsequent waves took place in 2002 (second wave), 2008 (third wave), 2011 (fourth wave), 2014 (fifth wave) and 2017 (sixth wave). Baseline samples were introduced in waves 2, 3 and 5 representing the DEAS study in a cohort-sequential design. In contrast, waves 4 and 6 were pure panel surveys. Therefore, most individuals were interviewed in wave 5 (10,324 individuals). This is why we restricted our analysis to this wave. More than 4000 individuals had already been interviewed in prior waves (response rate: 61%). Moreover, approximately 6000 participants were interviewed for the first time in wave 5 (response rate: 25%). After the interview, individuals could fill out a questionnaire that included more sensitive questions, such as life satisfaction, optimism, perceived autonomy, loneliness or perceived social isolation. In wave 5, 7952 individuals correctly filled out the drop-off questionnaires. Written informed consent was given by all participants. An ethics vote is not required in this study since the requirements for such a vote are not met (e.g., use of invasive methods).

### 2.2. Dependent Variables

Life satisfaction was quantified using the Satisfaction with Life Scale (SWLS) developed by Diener et al. [14], which has five items (in each case: five levels). The final score is expressed by the mean of the five items. Higher values indicate higher life satisfaction. Cronbach’s alpha was 0.86 in this study.

Optimism was measured using the Brandstädter and Wentura tool (1994), which has five items (in each case: four levels ranging from 1 = strong agreement to 4 = strong disagreement). The final score is calculated by taking the average score of the corresponding five items. Higher values are equivalent to higher optimism. Cronbach’s alpha was 0.85 in our study.

Perceived autonomy was quantified using the Schwarzer tool (2008), which has four items (in each case: four levels ranging from 1 = strong agreement to 4 = strong disagreement). The mean rating of all items was calculated. Higher values correspond to higher perceived autonomy. Cronbach’s alpha was 0.81 in our study.

Loneliness was quantified using the De Jong Gierveld tool (2006), which has six items (in each case: four levels ranging from 1 = strong agreement to 4 = strong disagreement). The index score was calculated by taking the average of all six items. Higher values indicate higher levels of loneliness. Cronbach’s alpha was 0.83 in our study.

Perceived social isolation was quantified using the Bude and Lantermann tool (2006), which has four items (in each case: 4 levels ranging from 1 (strong agreement) to 4 (strong disagreement)). The mean rating of all items was calculated. Higher values correspond to higher perceived social isolation. Cronbach’s alpha was 0.88 in our study. All tools used to quantify the outcomes are reliable and valid [15,16].

### 2.3. Independent Variables

In the DEAS survey, respondents were asked to identify, from a list of several illnesses, which illnesses they had been formally diagnosed with by their doctor. The occurrence of Parkinson’s (no; yes) was determined using responses to this section of the survey. The list of illnesses was determined in accordance with, among others, the Charlson Comorbidity Index [17] and supplementary consultations with specialists with a background in geriatrics.

In regression analysis, it was adjusted for several sociodemographic and health-related factors: sex (male; female), age (in years), educational level (ISCED-97) [18] (low education; medium education; high education), marital status (married, living together with spouse; married, living separated from spouse; divorced; widowed; single), employment status (working; retired; other; not employed), self-rated health (ranging from 1 = very good to 5 = very bad), and number of self-reported chronic conditions, including (i) cardiac and circulatory disorders, (ii) bad circulation, (iii) joint, bone, spinal or back problems, (iv) respiratory problems, asthma or shortness of breath, (v) stomach and intestinal problems, (vi) cancer, (vii) diabetes, (viii) gall bladder, liver or kidney problems, (ix) bladder problems, (x) eye problems or vision impairment, (xi) ear problems or hearing problems (count score, ranging from 0 to 11). In sensitivity analysis, the main regression model was extended by adding depressive symptoms as a covariate (using the 15-item Center for Epidemiologic Studies Depression Scale (CES-D) [19], ranging from 0 to 45, with higher values reflecting more depressive symptoms).

### 2.4. Statistical Analysis

First, sample characteristics were stratified by Parkinson’s disease (no; yes). Subsequently, effect sizes (Cohen’s d) were calculated for the associations between Parkinson’s disease and the psychosocial outcomes. Thereafter, multiple linear regressions were computed to investigate the association between Parkinson’s disease and the psychosocial outcomes, adjusting for sociodemographic and health-related factors.

The significance level was set at α = 0.05. Stata 17 was used to perform statistical analyses.

## 3. Results

### 3.1. Bivariate Analysis

The sample characteristics stratified by Parkinson’s disease are shown in Table 1. In our analytical sample, *n* equaled 7832 individuals (7777 individuals without Parkinson’s disease and 55 individuals with Parkinson’s disease). For example, average age was 64.4 years (SD: 11.2 years) among individuals without Parkinson’s disease, and it was 71.2 (SD: 9.7 years) among individuals with Parkinson’s disease. Moreover, 51.0% were female among individuals without Parkinson’s disease, whereas 32.7% were female among individuals with Parkinson’s disease.

With regard to psychosocial outcomes, while life satisfaction was 3.8 (SD: 0.7) among individuals without Parkinson’s disease, it was 3.6 (SD: 0.8) among individuals with Parkinson’s disease. Additionally, while perceived autonomy was 3.5 (SD: 0.5) among individuals without Parkinson’s disease, it was 3.0 (SD: 0.7) among individuals with Parkinson’s disease. Furthermore, while optimism was 3.0 (SD: 0.6) among individuals without Parkinson’s disease, it was 2.6 (SD: 0.7) among individuals with Parkinson’s disease. Moreover, while perceived social isolation was 1.6 (SD: 0.6) among individuals without Parkinson’s disease, it was 1.7 (SD: 0.7) among individuals with Parkinson’s disease. Finally, while loneliness was 1.8 (SD: 0.5) among individuals without Parkinson’s disease, it was 1.9 (SD: 0.6) among individuals with Parkinson’s disease. Further details are shown in Table 1.

In terms of effect sizes (Cohen’s d), the differences between individuals without Parkinson’s disease and individuals with Parkinson’s disease were as follows for the psychosocial outcomes: the association between Parkinson’s disease and perceived autonomy was d = 0.88; the association between Parkinson’s disease and optimism was d = 0.66; the association between Parkinson’s disease and life satisfaction was d = 0.27; the association between Parkinson’s disease and perceived social isolation was d = −0.22; the association between Parkinson’s disease and loneliness was d = −0.24.

### 3.2. Regression Analyis

The results of multiple linear regressions with psychosocial outcomes are displayed in Table 2. R^2^ values varied from 0.08 (with loneliness as outcome measure) to 0.19 (with life satisfaction as outcome measure). Adjusting for several covariates, regressions showed that individuals with Parkinson’s disease reported significantly lower perceived autonomy (β = −0.30, *p* < 0.01) compared to individuals without Parkinson’s disease. Moreover, regressions showed that individuals with Parkinson’s disease did not report worse psychosocial outcomes in terms of life satisfaction (β = −0.03, *p* = 0.77), loneliness (β = 0.08, *p* = 0.32), perceived social isolation (β = 0.02, *p* = 0.84) and optimism (β = −0.15, *p* = 0.07) compared to individuals without Parkinson’s disease.

In Table 3, the regression model was extended by adding depressive symptoms as a potential confounder. However, our results remained nearly the same. More precisely, after adjusting for several covariates, regressions showed that individuals with Parkinson’s disease reported significantly lower perceived autonomy (β = −0.28, *p* < 0.01) compared to individuals without Parkinson’s disease.

## 4. Discussion

### 4.1. Main Findings

Based on data from a large, nationally representative survey, our aim was to clarify the association between Parkinson’s disease and psychosocial factors. In terms of effect size, particularly large differences were identified between individuals with Parkinson’s disease and individuals without Parkinson’s disease with regard to perceived autonomy. Linear regressions showed that individuals with Parkinson’s disease reported significantly lower perceived autonomy compared to individuals without Parkinson’s disease. In contrast, they did not report worse psychosocial outcomes (in terms of life satisfaction, optimism, loneliness and perceived social isolation). Finally, while previous studies put emphasis on specific psychological factors such as depression, the current investigation extends that knowledge with a broader perspective on general psychosocial factors. By adding a control group (i.e., individuals without Parkinson’ s disease), our study markedly extends our current knowledge.

### 4.2. Relation to Previous Research

To date, only very few cross-sectional studies exist that explicitly investigated the link between Parkinson’s disease and psychosocial factors [20,21]. In general, our findings are difficult to compare with previous studies, as, unlike prior studies, our study included a control group (i.e., individuals without Parkinson’s disease) and former studies focused on different objectives. For instance, according to Nicolletti et al. [20], nonmotor symptoms are significantly associated with psychosocial well-being among individuals with Parkinson’s disease. This former study used the Psychological Well-being Scale as outcome measure. Another example: Cubo et al. [22] found that psychological factors were associated with life satisfaction among individuals with Parkinson’s disease. Nevertheless, it is worth repeating that these aforementioned studies failed to include a control group (individuals without Parkinson’s disease) and had divergent aims compared to our study.

Regarding our results, our study did not show an association between Parkinson’s disease and lower life satisfaction. This could be explained by habituation processes and, consequently, the adaptation to Parkinson’s disease and its motor and nonmotor symptoms. More precisely, former research showed individuals often have a (individual-specific) set-point of life satisfaction [23]. Thus, life events most often affect life satisfaction only in the short or midterm. In the long term, life satisfaction scores often bounce back to the individual-specific set-point [24]. We assume that Parkinson’s disease is such a critical life event that may temporarily affect life satisfaction, but individuals may adapt to Parkinson’s disease in the long term. However, future research is required to test this assumption. Moreover, perceived stigma may not be present. Additionally, according to our study, Parkinson’s disease is not associated with lower loneliness and lower perceived social isolation. This may be due to the lack of perceived stigma felt in daily life activities as, for example, in meetings with friends outside, restaurants visits in the public or outdoor sport activities. Due to the potentially absent perceived stigma, individuals with Parkinson’s disease have the courage to go out and participate in activities of daily living. That may be a potential explanation for the lower loneliness and perceived social isolation. However, there is very little knowledge regarding perceived stigma of Parkinson’s disease [25]. Thus, future research is urgently required to test our speculative hypotheses. Exploring the potential role of perceived stigma may assist in understanding a potential association between Parkinson’s disease and psychosocial factors.

Additionally, successful treatment outcomes (for example: supportive therapies, medication approaches or surgical interventions) could also play an important role in the explanation for the absent correlation. Moreover, Parkinson’s disease was not associated with lower optimism in our study. One way to explain this non-significant association is that individuals with Parkinson’s disease may experience trust for the current medicine and the coherent treatment success of the neurodegenerative disorder. However, it should be noted that this association was marginally significant (*p* < 0.10). Therefore, further research is required in this field to provide additional evidence.

Finally, our results demonstrated a significant association between Parkinson’s disease and lower perceived autonomy. This may occur due to the broad spectrum of support options, such as facilities for disabled individuals by the health care system or private medical establishments. Parkinson’s disease can easily lead to limitations in activities of daily living: for example, in the patient’s mobility in various daily situations, such as handling a phone, shopping or buying groceries, housekeeping and cleaning operations, laundry and washing activities. In addition to that, a possible health comparison with individuals in their age group (or comparisons with friends and relatives) may occur. More precisely, individuals with Parkinson’s disease may realize that, in contrast to themselves, other individuals in the same age bracket do not need such a level of additional help or support, for example, buying groceries or shopping. In that process, a potential increase in awareness (regarding their potential limitations) may take place. Therefore, individuals with Parkinson’s disease may report a lower perceived autonomy—compared to individuals without Parkinson’s disease. However, future research is needed to test our assumptions.

### 4.3. Strengths and Limitations

A major strength of our study is that we used data from a large, nationally representative population sample of community-dwelling individuals aged 40 and over in Germany. Additionally, well-validated scales were used to quantify the psychosocial factors. Physician-diagnosed Parkinson’s disease was used as the explanatory variable. In contrast to the large majority of prior studies, we also included individuals without Parkinson’s disease (control group). Moreover, this is one of the first studies to investigate the association between Parkinson’s disease and psychosocial factors. However, our study also has some limitations. Only a small number of individuals had Parkinson’s disease. Additionally, a small sample selection bias in the DEAS study should be noted. Therefore, it could be difficult to generalize the study findings to individuals with impaired German language skills or to individuals with a low educational level. Due to the cross-sectional design, clarifying the directionality between Parkinson’s disease and our psychosocial outcomes is difficult. Additionally, the association between Parkinson’s disease and psychosocial factors may vary depending on country-specific characteristics, such as the availability of psychotherapeutic/psychosocial support and, for example, national health services and social and cultural obstacles to the health care system. Moreover, our data are from the year 2014. In future research, it would be interesting to examine the association between Parkinson’s disease and psychosocial factors with more recent data (e.g., during the COVID-19 pandemic). Furthermore, the missing associations between Parkinson’s disease and psychosocial factors (such as life satisfaction) may be explained by the possibly low severity of Parkinson’s disease or by the duration of diagnosis. It should also be acknowledged that the severity of PD symptoms (e.g., by using UPDRS-III [26] or Hoehn and Yahr) [27] was not quantified in our study. Thus, future research in this area is urgently required.

## 5. Conclusion and Future Research

Study findings showed a quite strong association between Parkinson’s disease and perceived autonomy. Surprisingly, we did not identify an association between Parkinson’s disease and other psychosocial factors, such as loneliness or satisfaction with life. This knowledge is important for, among other things, general practitioners, neurologists, professional caregivers and spousal or other informal caregivers, as well as other relatives and friends of individuals with Parkinson’s disease.

Future research could elucidate the underlying mechanisms in the association between Parkinson’s disease and perceived autonomy. Factors such as internal or external locus of control may be of importance in this association. For example, individuals with a high internal locus of control believe that life’s outcomes are based on their own efforts, whereas individuals who score high in external locus of control believe that outcomes are based on external factors such as fate. Individuals with Parkinson’s disease who have a high internal locus of control may modify their lifestyle to maintain autonomy for as long as possible. Moreover, future studies are required to investigate the association between Parkinson’s disease and psychosocial factors in nursing homes or old age homes. Additionally, further subgroup analyses (e.g., stratified by sex, age group, severity of Parkinson’s disease) should be conducted in future studies. Additionally, upcoming studies should examine the long-term psychosocial impact of Parkinson’s disease. Beyond that, psychosocial factors may become of future interest, as they may be important modulators of motor (sequence) learning in individuals with Parkinson’s disease [28]. Such modulators can be positive task emotions and increased general self-efficacy. In this regard, the role of social interaction in motor skill learning, as well as the role of mindset and self-regulatory mechanisms, in Parkinson’s disease patients is yet not fully identified. Thus, it must be incorporated and investigated in detail, as it could lead to the development of enhanced non-pharmacological interventions intended to preserve motor function and reduce unpleasant psychosocial effects [28].

## Figures and Tables

**Table 1:** Sample characteristics stratified by Parkinson’s disease (*n* = 7832).

**Variables**	**Individuals** **without** **Parkinson’s** **Disease**	**Individuals with Parkinson’s Disease**
	*n* = 7777	*n* = 55
**Perceived autonomy** (ranging from 1 to 4, with higher values representing high perceived autonomy)	3.5 (0.5)	3.0 (0.7)
**Optimism** (ranging from 1 to 4, with higher values representing high optimism)	3.0 (0.6)	2.6 (0.7)
**Life satisfaction** (ranging from 1 to 5, with higher values representing higher life satisfaction)	3.8 (0.7)	3.6 (0.8)
**Perceived social isolation** (ranging from 1 to 4, with higher values representing higher perceived social isolation)	1.6 (0.6)	1.7 (0.7)
**Loneliness** (ranging from 1 to 4, with higher values representing higher loneliness)	1.8 (0.5)	1.9 (0.6)
**Sex**		
1. male	3808 (49.0%)	37 (67.3%)
2. female	3969 (51.0%)	18 (32.7%)
**Age in years**	64.4 (11.2)	71.2 (9.7)
**Educational level** (ISCED-97 classification)		
1. low (ISCED 0–2)	507 (6.5%)	9 (16.4%)
2. medium (ISCED 3–4)	4007 (51.5%)	25 (45.5%)
3. high (ISCED 5–6)	3263 (42.0%)	21 (38.2%)
**Marital status**		
1. married, living together with spouse	5436 (69.9%)	41 (74.5%)
2. married, living separated from spouse	125 (1.6%)	2 (3.6%)
3. divorced	787 (10.1%)	2 (3.6%)
4. widowed	875 (11.3%)	7 (12.7%)
5. single	554 (7.1%)	3 (5.5%)
**Employment status**		
1. working	2846 (36.6%)	8 (14.5%)
2. retired	4229 (54.4%)	45 (81.8%)
3. not employed	702 (9.0%)	2 (3.6%)
**Self-rated health** (from 1 = very good to 5 = very bad)	2.5 (0.8)	3.4 (0.9)
**Number of chronic diseases** (ranging from 0 to 11)	2.6 (1.9)	3.1 (2.1)

**Table 2:** Determinants of psychosocial factors. Results of multiple linear regressions.

	Perceived Autonomy	Optimism	Life Satisfaction	Perceived Social Isolation	Loneliness
Presence of Parkinson’s disease (Ref.: Absence of Parkinson’s disease)	−0.30 ** (0.09)	−0.15 + (0.08)	−0.03 (0.10)	0.02 (0.09)	0.08 (0.08)
Potential confounders	✓	✓	✓	✓	✓
*R* ^2^	0.10	0.18	0.19	0.10	0.08
Observations	7803	7832	7791	7764	7738

Unstandardized beta-coefficients are reported; robust standard errors in parentheses; ** *p* < 0.01, + *p* < 0.10; potential confounders include sex, age, educational level, marital status, employment status, self-rated health, number of chronic diseases. Therefore, we used the “✓” symbol.

**Table 3:** Determinants of psychosocial factors. Results of multiple linear regressions (additionally adjusting for depressive symptoms).

	Perceived Autonomy	Optimism	Life Satisfaction	Perceived Social Isolation	Loneliness
Presence of Parkinson’s disease (Ref.: Absence of Parkinson’s disease)	−0.28 ** (0.09)	−0.11 (0.08)	0.01 (0.10)	−0.01 (0.09)	0.05 (0.08)
Potential confounders	✓	✓	✓	✓	✓
*R* ^2^	0.12	0.27	0.25	0.15	0.15
Observations	7800	7829	7788	7761	7735

Unstandardized beta-coefficients are reported; robust standard errors in parentheses; ** *p* < 0.01; potential confounders include sex, age, educational level, marital status, employment status, self-rated health, number of chronic diseases and depressive symptoms. Therefore, we used the “✓” symbol.

## Data Availability

The data used in this study are third-party data. The anonymized data sets of the DEAS (1996, 2002, 2008, 2011, 2014, 2017 and 2020) are available for secondary analysis. The data have been made available to scientists at universities and research institutes exclusively for scientific purposes. The use of data is subject to written data protection agreements. Microdata of the German Ageing Survey (DEAS) are available free of charge to scientific researchers for non-profitable purposes. The FDZ-DZA provides access and support to scholars interested in using DEAS for their research. However, for reasons of data protection, signing a data distribution contract is required before data can be obtained. For further information on the data distribution contract, please see https://www.dza.de/en/research/fdz/access-to-data/formular-deas-en-english (accessed on 1 July 2022).
